# Binding of TCR Multimers and a TCR-Like Antibody with Distinct Fine-Specificities Is Dependent on the Surface Density of HLA Complexes

**DOI:** 10.1371/journal.pone.0051397

**Published:** 2012-12-10

**Authors:** Jianrong L. Low, Anneta Naidoo, Gladys Yeo, Adam J. Gehring, Zi Zong Ho, Yin Hoe Yau, Susana G. Shochat, David M. Kranz, Antonio Bertoletti, Gijsbert M. Grotenbreg

**Affiliations:** 1 Singapore Institute for Clinical Sciences, Agency for Science Technology and Research, (A*STAR), Singapore, Singapore; 2 Department of Biochemistry, University of Illinois at Urbana-Champaign (UIUC), Urbana, Illinois, United States of America; 3 Department of Microbiology, Yong Loo Lin School of Medicine, National University of Singapore (NUS), Singapore, Singapore; 4 Department of Biological Sciences, Yong Loo Lin School of Medicine, National University of Singapore (NUS), Singapore, Singapore; 5 Department of Medicine, Yong Loo Lin School of Medicine, National University of Singapore (NUS), Singapore, Singapore; 6 Immunology Programme, Yong Loo Lin School of Medicine, National University of Singapore (NUS), Singapore, Singapore; 7 Nanyang Technological University (NTU), School of Biological Sciences, Singapore, Singapore; Massachusetts General Hospital, United States of America

## Abstract

Class I Major Histocompatibility Complex (MHC) molecules evolved to sample degraded protein fragments from the interior of the cell, and to display them at the surface for immune surveillance by CD8^+^ T cells. The ability of these lymphocytes to identify immunogenic peptide-MHC (pMHC) products on, for example, infected hepatocytes, and to subsequently eliminate those cells, is crucial for the control of hepatitis B virus (HBV). Various protein scaffolds have been designed to recapitulate the specific recognition of presented antigens with the aim to be exploited both diagnostically (*e.g.* to visualize cells exposed to infectious agents or cellular transformation) and therapeutically (*e.g.* for the delivery of drugs to compromised cells). In line with this, we report the construction of a soluble tetrameric form of an αβ T cell receptor (TCR) specific for the HBV epitope Env_183–191_ restricted by HLA-A*02:01, and compare its avidity and fine-specificity with a TCR-like monoclonal antibody generated against the same HLA target. A flow cytometry-based assay with streptavidin-coated beads loaded with Env_183–191_/HLA-A*02:01 complexes at high surface density, enabled us to probe the specific interaction of these molecules with their cognate pMHC. We demonstrate that the TCR tetramer has similar avidity for the pMHC as the antibody, but they differ in their fine-specificity, with only the TCR tetramer being capable of binding both natural variants of the Env_183–191_ epitope found in HBV genotypes A/C/D (187Arg) and genotype B (187Lys). Collectively, the results highlight the promiscuity of our soluble TCR, which could be an advantageous feature when targeting cells infected with a mutation-prone virus, but that binding of the soluble oligomeric TCR relies considerably on the surface density of the presented antigen.

## Introduction

Antibodies and T cell receptors (TCRs) represent two distinct classes of immune molecules that the adaptive immune system in mammals has evolved to recognize foreign pathogens. Whereas antibodies can function as soluble molecules, TCRs are found only as membrane bound receptors [1]. Furthermore, while antibodies are able to recognize antigens alone, often as linear or conformational epitopes of a polypeptide, TCR recognition invariably requires the antigenic peptide to be presented by a major histocompatibility complex (MHC) product on the cell surface [2]. For example, peptide-MHC (pMHC) complexes are formed in virus-infected cells when processed viral proteins are loaded onto class I MHCs and delivered to the cell surface [3]. This enables infected cells to be recognized, typically by a CD8^+^ T cell, resulting, ideally, in activation of the T cell, destruction of the target cell, and clearance of the virus [4]. In hepatitis B virus (HBV) infection, which still poses a global health problem despite the availability of an effective prophylactic vaccine, CD8^+^ cytotoxic T lymphocytes (CTLs) play a crucial role in the antiviral response [5]. Effective control of HBV relies on robust CTL responses, and CD8^+^ T cell dysfunction is associated with chronic infection, which in turn carries the risk of complications such as hepatocellular carcinoma.

The ability to activate CTLs and the magnitude of CTL responses correlates with the levels and density of pMHC presented by HBV-infected cells [6]. This has raised an interest to both visualize the presented antigens on the surface of infected hepatocytes, and to generate molecular entities that enable the targeted delivery of pharmaceutical or immunomodulatory compounds [7], [8]. A promising approach uses monoclonal antibodies (mAbs) with TCR-like specificities for distinct pMHC products, which attain affinities higher than normal TCRs [7], [9], [10]. For such novel TCR-like mAbs, however, it remains important to individually assess whether they exhibit the same specificity as the cognate TCRs [11], [12]. Structural studies already demonstrated that the docking position could differ from the conventional TCR, exhibiting interactions predominantly with the MHC, thus affecting peptide specificity [13]. In addition, TCR-like mAbs are often raised in non-human species (*e.g.* mouse, rat or rabbit) and require subsequent transformation into human chimeras; a process known as “antibody humanization”, to be of therapeutic value. Because such mAbs have not been selected *in vivo* in the target species, cross-reactivity with self-antigens can also formally not be ruled out.

An alternative strategy employs multivalent soluble TCRs to target antigens presented on the cell surface [14], [15], analogously to probing clonotypically expressed TCRs with oligomeric pMHC molecules, which are routinely used for the identification and characterization of virus-specific CTLs [16], [17]. For successful implementation, however, several technical limitations need to be addressed. First, the expression and production of soluble TCRs is often problematic [18]. Second, the affinities of TCRs are normally in the micromolar range, making detection of pMHC on cell surfaces with a soluble TCR probe difficult [19]. Third, while the TCR is expressed on T cells at a uniform, relatively high density (about 50,000 molecules per cell; [20]) the number of naturally processed antigenic pMHC complexes per cell is thought to be low, in the range of 10 to 1000 [21]. To overcome these challenges, soluble TCRs have been recombinantly produced in various systems, such as insect cells [22] and *E. coli* [23]–[26]. The low affinities of the monomeric TCRs have been enhanced both by oligomerization [14], [15], [27]–[29], and by directed evolution in either yeast or phage display systems [30], [31], or by rational design [32], [33].

In an effort to develop novel protein constructs for diagnostic and therapeutic application in the context of HBV, we generated a soluble form of an αβ T cell receptor isolated from an HLA-A*02:01 restricted CD8^+^ T cell clone specific for the immune-dominant Env_183–191_ epitope (Env183/A2) of the HBV envelope protein. We show that the soluble TCR is both functional and highly peptide-specific using a multivalent, bead-based assay that we developed as a novel platform for probing TCR:pMHC interactions. Using this assay, we compared the Env_183–191_ peptide fine-specificity of both the TCR and a monoclonal antibody previously isolated to recognize the same Env_183–191_/HLA-A*02:01 [7]. Finally, we established that the surface density of HLA complexes is a critical factor when TCR multimers and TCR-like mAbs are employed to detect MHC-presented peptide antigens.

## Materials and Methods

### Cloning of TCR α and β Chains

The Vα34 and Vβ28 Env183/A2 specific heterodimeric TCR was previously isolated from a T cell clone [34]. Each chain was truncated, the α chain after Ser202 and the β chain after Asp243, and cloned into pET28a expression vectors separately by PCR using 5′ primers containing an NcoI restriction enzyme site that also contains the ATG start codon and 3′ primers that contains the stop codons and an XhoI restriction enzyme site. A disulfide bond was introduced by mutating residues Thr48 of the α-chain constant domain and Ser57 of the β-chain constant domain into Cys residues (as described previously [23]). In addition, an unpaired Cys75 of the β-chain constant domain was mutated to a serine to facilitate the specific pairing of the introduced Cys residues. A biotinylation motif was added to the C-terminal end of the β-chain to facilitate tetramerization with streptavidin (Fig. S1).

### Expression of TCR Chains

The TCR chains were separately expressed in Rosetta™ Competent *Escherichia coli* cells (Novagen). Inclusion bodies were harvested from cells lysed by sonication and washed using washing buffer (50 mM Tris, 100 mM NaCl, 0.5% Triton X-100, 1 mM EDTA, 0.1% sodium azide). 10 mg/L of biotin was added during induction to the β chain expression cultures to facilitate *in vivo* biotinylation. Inclusion pellets were then dissolved completely in urea solution (8 M urea, 25 mM MES, 10 mM EDTA, 0.1 mM DTT, pH 6.0). Solubilized inclusion bodies were quantitated by Bradford assay (Biorad, Richmond, California) and stored at −80°C.

### Refolding and Purification of Refolded TCRs

The soluble TCR was refolded by rapidly diluting aliquots of 10 mg of each chain in 1 litre of refold buffer (100 mM Tris pH 8.0, 400 mM L-Arg HCl, 2 mM EDTA, 5 mM reduced glutathione, 50 mM oxidized glutathione) supplemented with a tablet of cOmplete ULTRA protease inhibitor cocktail (Roche Applied Science). Two further injections of each inclusion bodies 8 hrs apart yielded a final concentration of 60 mg/mL total protein. The refolded protein was concentrated using a Vivaflow 50 crossflow cassette (Sartorius Biotech) and dialyzed twice into 2.5 litres of 20 mM Tris pH 8.0 overnight. Enzymatic biotinylation was carried out according to manufacturer’s instructions using BirA biotin ligase (Avidity). Purification of biotinylated TCRs was carried out by size exclusion chromatography (HiPrep 16/60 Sephacryl S200) followed by anion exchange chromatography (MonoQ 5/50 GL) using an AktaFPLC system. Fractions containing refolded heterodimeric TCRs were identified by SDS-PAGE. Biotinylation of purified refolded TCR was probed by gel shift assay whereby refolded TCR prepared in non-reducing SDS-PAGE loading buffer were incubated with excess streptavidin at room temperature (RT) for 15 mins before SDS-PAGE analysis.

### Multimerization of Refolded TCRs

Soluble TCRs were tetramerized by the addition of allophycocyanin (APC) conjugated-streptavidin (Invitrogen) to the monomers to a final volume of 1∶4 respectively. The total amount of streptavidin-APC required was divided into 5 aliquots and added to TCR monomers in 30 mins interval. TCR tetramers were then stored at 4°C and used within a month.

### TCR-like Antibody

A mouse monoclonal antibody specific for the HLA-A*02:01-restricted Env_183–191_ epitope was previously generated [7]. Antibodies were purified from the supernatant of the hybridoma culture using a protein G-agarose column, concentrated and quantified by Bradford Assay (Biorad, Richmond, California).

### Peptide-MHC Conjugated Beads Assay

Fluorescent streptavidin-coated yellow particles (Spherotech SVFA-2552-6K and SVFB-2552-6K), or non-fluorescent streptavidin-coated particles (Spherotech SVP-30-5), with an average diameter of ∼2.7 µm, were chosen as scaffolds for the immobilization of high numbers of specific pMHCs. To generate the pMHC-conjugated beads, HLA-A*02:01 was first recombinantly expressed as inclusion bodies in an *Escherichia coli* system, refolded *in vitro* with the conditional ligand GILGFVF-J-L, then biotinylated enzymatically and purified as previously described [35], [36]. UV-mediated peptide exchange of these caged pMHC molecules, following established procedures [35]–[38], produced pMHCs loaded with the peptide of interest. 20 µL of streptavidin beads was pre-incubated in 200 µL of blocking buffer (2% BSA in PBS) for 30 min with shaking. 25 µL of peptide-exchanged pMHC (0.5 µM) was then added and the beads were incubated for 1 h with shaking at RT. The array of beads presenting various densities of pMHC was generated by titrating pMHC in 4-fold dilutions in blocking buffer before conjugation. The beads were pelleted by 5 min centrifuging at 800 *g* and washed with 200 µL blocking buffer. Staining of the beads was then done with either TCR tetramers (1 µg/mL) or TCR-like antibody (1 µg/mL). Staining of the conjugated beads was done at RT with shaking for 30 min, followed by washing with 200 µL of blocking buffer, fixation with 200 µL of 1% paraformaldehyde in PBS, and flow cytometry analysis. Detection of TCR-like antibody binding was accomplished with an APC-conjugated goat anti-mouse antibody (Invitrogen) at RT with shaking for 20 min. Data acquisition was performed on an LSR-II or FACSCanto flow cytometer (Becton Dickinson), followed by analysis using FlowJo software (Tree Star) and GraphPad Prism software (GraphPad).

### Cell Culture and Staining

T2 cells (ATCC CRL 1992) and EBO-PreS1 cells [39] were cultured in RPMI 1640 medium while HepG2.2.15 cells were cultured in Dulbecco’s modified Eagle’s medium, both supplemented with 10% fetal bovine serum (FBS), 20 mM HEPES, 0.5 mM sodium pyruvate, minimal essential medium (MEM), nonessential amino acids, Glutamax, 5 µg/mL Plasmocin (InvivoGen), 100 U/mL penicillin, and 100 µg/mL streptomycin. Cells were treated with 100 U/mL IFN-γ (R&D systems) for 24 hrs before washing and pulsing. Cells were pulsed with 1 µM or 10 µM final concentration of peptides at RT before staining. Washing and staining were done in flow cytometry buffer (1% BSA, 0.01% sodium azide, in PBS). Cells were stained with 1 ug/mL of Env183/A2 antibody or TCR tetramer unless otherwise stated at RT for 30 mins with shaking. Secondary detection of TCR-like antibody was done with an APC conjugated goat anti-mouse antibody (Invitrogen).

### Surface Plasmon Resonance

Binding studies were carried out using the BIAcore™ 3000 (BIAcore AB, GE Healthcare). Neutravidin was immobilized onto a CM5 sensor chip by standard amine coupling procedure. Briefly, the surface was activated for 5 min with 1∶1 mixture of 0.2 M N-ethyl-N’-[3-(diethylamino)propyl]carbodiimide (EDC) and 50 mM N-hydroxysuccinimide (NHS). Neutravidin was dissolved into 10 mM sodium acetate at pH 5.0 to a final concentration of 50 µg/mL and injected across the activated surface at 10 µL/min to achieve 3000 RU. The surface was finally blocked by injection of 1 M ethanolamine at pH 8.5 for 7 min. Next, 10 µM biotinylated TCR monomers were captured by neutravidin-biotin chemistry to saturation followed by a blocking step using 5 mM biotin for 6.5 min. Equilibrium affinity was determined at 25°C in running buffer (50 mM Tris, 150 mM NaCl, pH 7.0). Soluble Env183/A2 pMHC monomers or control Core18/A2 pMHC monomers (in a 3-fold dilution series with a highest concentration of 10 µM) were flowed over the immobilized TCR monomers at 30 µL/min for 2 min and then allowed to dissociate for 5 min. The results were analysed with BIAevaluation software (BIAcore AB, GE Health). Binding responses were corrected by subtracting the responses of the Core18/A2 pMHC control from those of the Env183/A2 pMHC. The equilibrium dissociation constant K_D_ was then determined by plotting the equilibrium binding responses against pMHC concentrations and fit to a steady-state model.

### Structural Modeling

The Env_183–191_ peptide was modeled into the Tax-HLA-A*02:01 (PDB-ID:1DUZ) using the Rosetta Backrub program (http://kortemmelab.ucsf.edu) [40]. Figures were made using Pymol (DeLano Scientific Research LLC).

## Results

### Producing Soluble Env_183–191_ Peptide/HLA-A*02:01-specific TCR Tetramers

**Figure 1:**
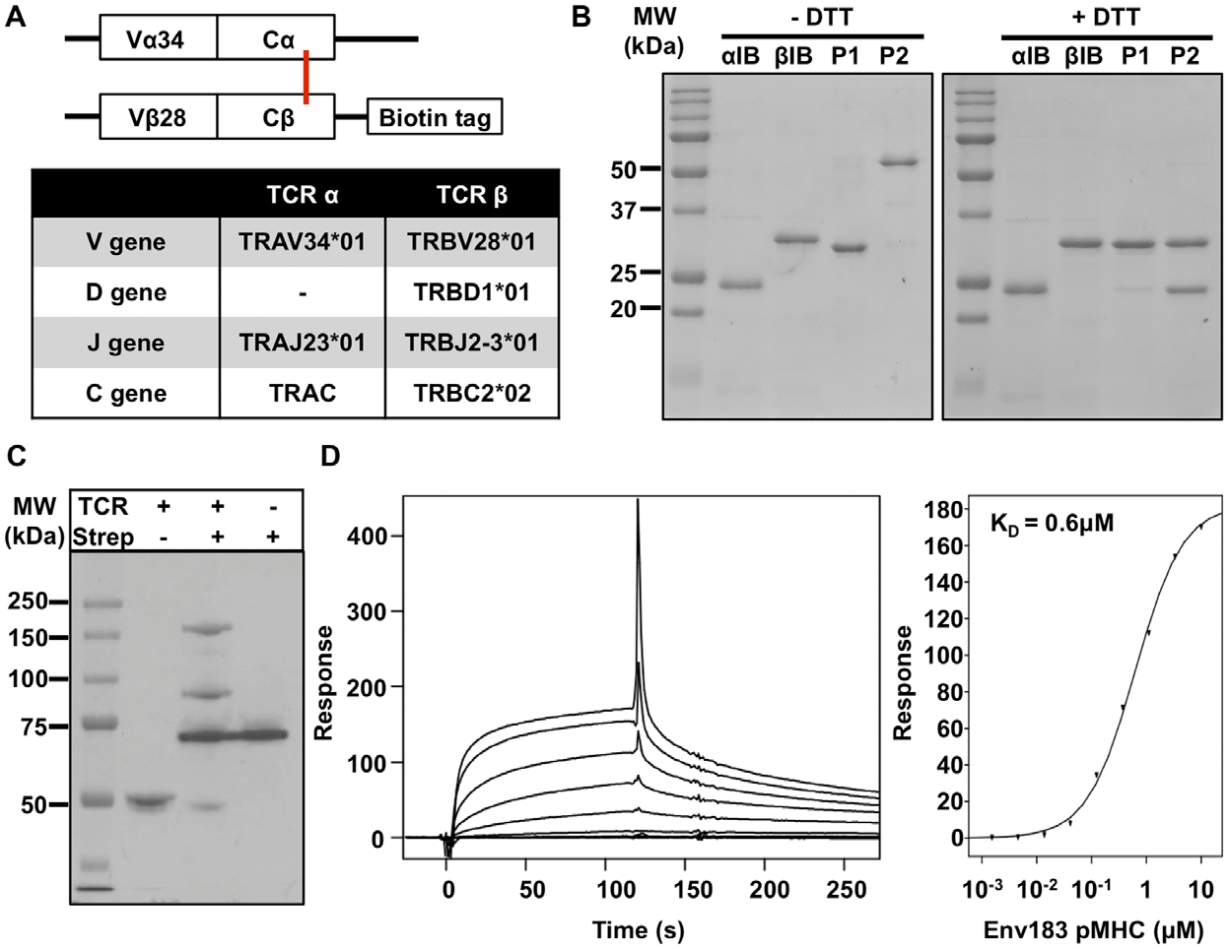
Expression and purfication of the Env183/A2 soluble T cell receptor. (A) The gene encoding for TCR α chain residues 1–202 and TCR β chain residues 1–243 were separately cloned into pET28a vectors. A BirA recognition sequence was added to the C-terminus of the β chain. Cα Thr48Cys and Cβ Ser57Cys mutations were introduced to facilitate an inter-chain disulfide bond (red bar). (B) SDS PAGE analysis of two distinct fractions obtained after chromatographic purification of refolded TCR. (C) Gel shift analysis of the purified TCR shows >90% biotinylation. (D) Surface plasmon resonance analysis of refolded TCR monomers demonstrated functional binding to immobilized Env183/A2 with a K_D_ of 0.6 µM, as determined with a steady-state model.

The genes encoding the extracellular regions of the α and β chains of the Env183/A2 TCR [34], with an additional cysteine introduced in each C region [23] (Fig. 1A), were cloned into pET28a vectors and the proteins were expressed in *E. coli* as inclusion bodies. The soluble α and β chains have predicted molecular weights of ∼23 kDa and ∼28 kDa, respectively. Equal amounts of inclusion bodies were combined under high dilution, and the concentrated solution was subjected to size exclusion chromatography (Fig. S2A). A peak corresponding to the expected size of the heterodimeric TCR (∼51 kDa) was subjected to anion exchange chromatography (Fig. S2B). Fractions corresponding to observed peaks P1 and P2 were pooled, concentrated and analyzed by SDS-PAGE under reducing and non-reducing conditions (Fig. 1B). P1 appeared to contain predominantly β chain presumably representing soluble non-disufide linked β chain refolded monomers. The increased electrophoretic mobility under non-reducing SDS-PAGE of this fraction, compared to the inclusion body preparation, we attribute to intramolecular disulfide bonding. In contrast, P2 had the anticipated equal stoichiometry of α and β chain, corresponding to the disulfide-linked αβ heterodimer. All subsequent studies were performed with this complex. To generate multivalent forms of the TCR, a BirA biotin tag was included at the C terminus of the β chain (Fig. 1A). To assess if the purified protein (P2) had been biotinylated during recombinant expression, gel shift analysis was performed by incubation with strepavidin (Fig. 1C). This showed that the majority of the product “gel-shifted” which is indicative of biotinylation. Fluorescent TCR tetramers were produced by incubation with streptavidin-allophycocyanin (SA-APC) in a 4 to 1 stoichiometry. Lastly, the functionality of the refolded soluble TCR was assessed with surface plasmon resonance by injecting Env183/A2 pMHC over the TCR monomers immobilized on a sensor chip (Fig. 1D). Analysis of equilibrium binding, using a steady-state model, established an affinity (K_D_) of 0.6 µM for the Env183/A2 specific TCR.

### A Bead-based Assay to Probe pMHC Binding to Soluble TCR Tetramers

**Figure 2:**
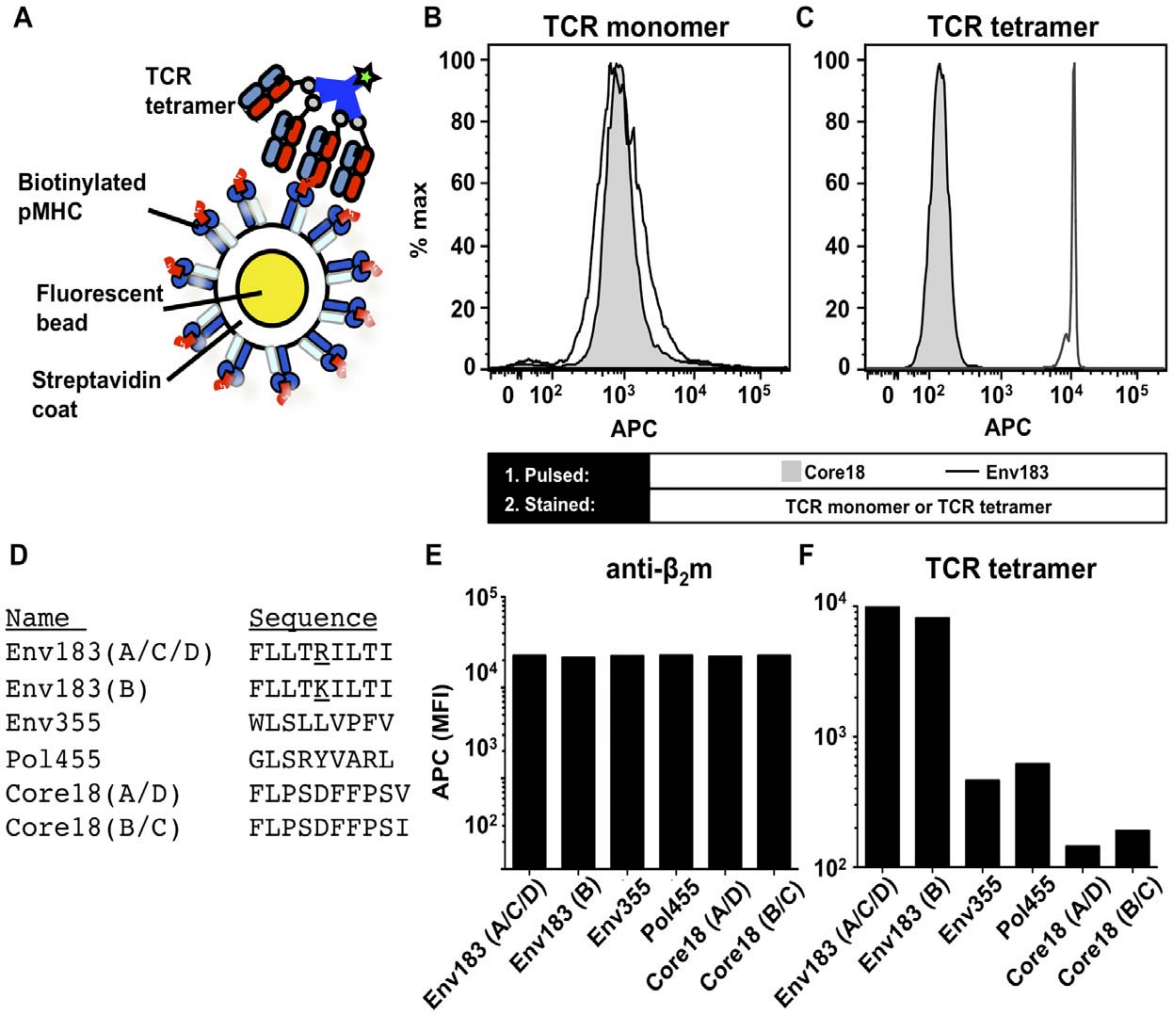
pMHC-coated beads as an artificial antigen presenting surface. (A) Schematic of the fluorescent bead assay. Streptavidin-coated beads were loaded with biotinylated pMHC monomer thus providing a homogenous binding surface for the Env183/A2 mAb or TCR tetramers. (B) Beads were stained with 10 µg/mL of monomeric TCRs or (C) 1 µg/mL APC-conjugated TCR tetramers. Beads presenting Core_18–27_ HLA-A*02:01 were used as a control. (D) Beads were loaded with HLA-A*02:01 presenting 6 different peptides and stained with a mouse anti-β2m followed by detection with an APC-conjugated goat anti-mouse antibody. (E) Staining shows equal levels of pMHC on beads. (F) The beads were furthermore stained with 1 µg/mL APC-conjugated TCR tetramers, showing that they only bound Genotype A/C/D and Genotype B variants of Env_183–191_ peptides presented by HLA-A*02:01.

We explored an additional system to characterize the binding of soluble TCR and used streptavidin-coated beads conjugated with biotinylated soluble pMHC molecules (Fig. 2A) to provide a homogenous target with a high ligand density [37]. Recombinant biotinylated HLA-A*02:01 was first refolded with a conditional ligand. Peptide-exchange was accomplished by first UV-irradiating the refolded pMHC in the presence of Env_183–191_ or Core_18–27_ (control) peptide [36], [38], [41]–[43]. The pMHC molecules with the desired specificity were then immobilized on the beads and stained with either TCR monomers, followed by an APC-conjugated secondary detection Ab, or TCR tetramers (Fig. 2B,C). Monomer binding was below the detection limit, but TCR tetramer staining of the Env183/A2-coupled beads was increased by two orders of magnitude (Fig. 2C) compared to beads coated with HLA-A*02:01 presenting the Core_18–27_ peptide. This demonstrated that the bead assay provides a sensitive system for examining the specificity of soluble TCRs. Consequently, different epitopes derived from the hepatitis B virus envelope, core and polymerase proteins, including two natural variants of the Env_183–191_ epitope (Fig. 2D) were peptide-exchanged into HLA-A*02:01 complexes and conjugated to the streptavidin-coated beads. The levels of beta-2-microglobulin (β2m), indicative of MHC stability, showed that the number of pMHC molecules present was approximately equal for all pMHC products (Fig. 2E). The TCR tetramer bound specifically both Env_183–191_ peptide variants derived from two viral genotypes but not to the other HBV epitopes restricted by HLA*02:01 (Fig. 2F). The Env_183–191_ epitope variants are found in either genotype A/C/D or genotype B viral strains and the TCR thus provides pan-specific recognition. This promiscuity is consistent with activation data of transduced T cells expressing the same α and β TCR genes [7].

### TCR Tetramers and the Analogous TCR-like mAb have Distinct Fine-specificities

**Figure 3:**
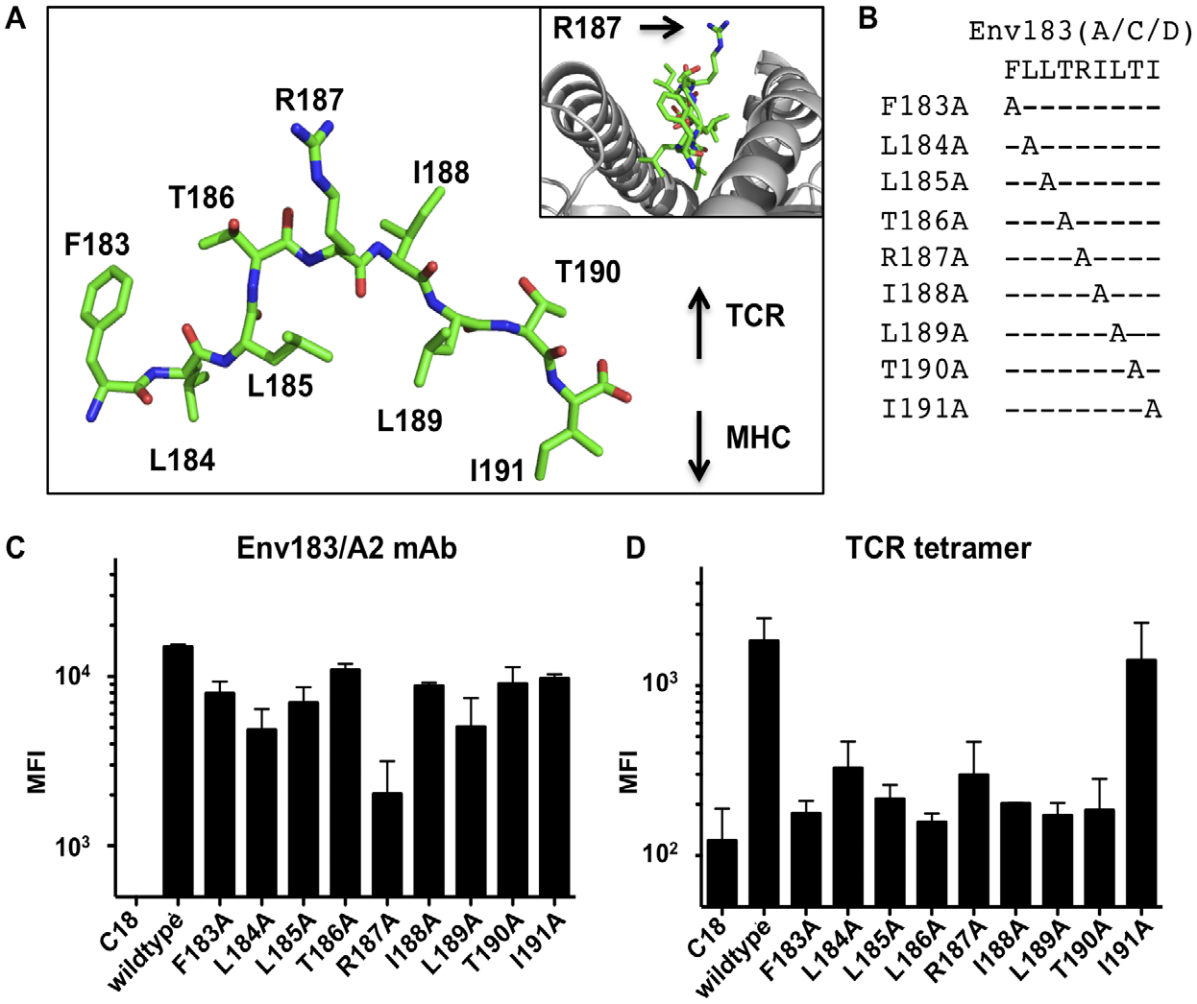
The fine-specificity of an Env183/A2 mAb and soluble TCR tetramers. (A) The Env_183–191_ peptide modeled into the Tax/HLA-A2 crystal structure, inset: lateral view of the peptide in HLA-A*02:01. The Arg187 side chain protrudes from the binding pocket, potentially interacting with the TCR or antibody. (B) Nine different Env_183–191_ peptides with single position alanine substitutions were used to probe the fine specificity. (C and D) The alanine variant pMHCs were loaded onto beads and stained with Env183/A2 mAb or TCR tetramer (1 µg/mL).

It has been reported that a TCR-like monoclonal antibody (mAb) that recognizes the composite surface of HLA-A*02:01 presenting the Env_183–191_ epitope (Env183/A2 mAb) was capable of binding only genotype A/C/D variants of the peptide epitope and not the genotype B variant [7]. The difference between these two peptides lies in a single conserved amino acid at position 187 (Lys for genotype B and Arg for genotype A/C/D, Fig. 2D). Modeling studies revealed that the arginine side chain points away from HLA-A*02:01, and is solvent exposed, making it available for TCR interaction (Fig. 3A). To experimentally determine the fine-specificity, individual alanine substitutions at every position of the Env_183–191_ peptide were generated and loaded into caged HLA-A*02:01 (Fig. 3B), which were then conjugated to beads and probed with either the TCR tetramers or Env183/A2 mAb. The levels of β2m were probed, showingthat the number of pMHC molecules on the surface was approximately equal for all pMHC products (Fig. S3). Alanine substitution at position 187 had the largest impact on Env183/A2 mAb binding among all the alanine variants (Fig. 3C). While the TCR tetramer was able to tolerate the conservative amino acid variation between lysine and arginine (Fig. 2F), alanine substitutions had substantially reduced TCR tetramer binding (Fig. 3D). TCR tetramer binding appeared to be affected by alanine substitutions at all positions except the C-terminus. This observation agrees with T-cell activation data with transduced T cells expressing the same αβ TCR [7]. The differences in binding to the presented peptide variants imply that although the two molecules bind the same pMHC ligand, they are likely to have different pMHC docking positions and/or different chemistries of interaction.

### Detection Limit of the Bead-based Assay

**Figure 4:**
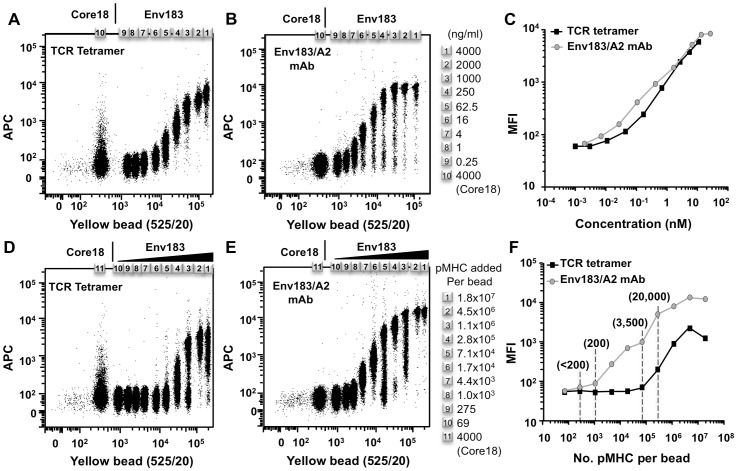
Functional avidity of the Env183/A2 mAb and soluble TCR tetramers. A range of beads of different fluorescent intensities were saturated with pMHC and stained with titrating amounts of (A) TCR tetramer or (B) Env183/A2 mAb. (C) The titration profiles of the TCR tetramer and the Env183/A2 mAb showed that both reagents had similar avidity for the pMHC. An array of beads presenting pMHCs at different densities was generated, as confirmed and quantified by anti-β2m staining (Figure S4). These beads were incubated with 1 µg/mL of (D) TCR tetramer or (E) Env183/A2 mAb. (F) Graphical representation of staining intensity for the different bead populations. Whereas the antibody bound beads presenting 200 complexes, the TCR tetramer required at least 3500 complexes for similar staining.

To determine the sensitivity of our assay, we titrated the soluble TCR tetramer, the mAb, and the amount of Env183/A2 complexes on beads. We multiplexed the system by employing a series of streptavidin-coated beads with varying fluorescent intensities. To examine the optimal concentration of TCR or mAb required for binding, the collection of beads were individually coated with saturating amounts of the Env183/A2 complexes. A titration of TCR tetramer demonstrated that TCR tetramers were able to bind their cognate pMHC at concentrations as low as 10 ng/mL (0.04 nM) (Fig. 4A). The same collection of Env183/A2 strepavidin-coated beads was stained with the Env183/A2 mAb (Fig. 4B). Comparison of these titrations showed that at the same protein concentration both TCR tetramer and Env183/A2 mAb had similar avidity for the Env183/A2 products (Fig. 4C). Subsequently, the influence of pMHC density on binding was examined with a fixed concentration of soluble TCR tetramer or Env183/A2 mAb. The bead populations were loaded with varying amounts of Env183/A2, providing an array of beads presenting pMHC at different densities. Staining with anti-β2m verified the decreasing amounts of pMHC on the surface (Fig. S4B), and allowed us to quantify the number of pMHC per bead (Fig. S4C). This revealed that, at saturation, the beads displayed approximately 80,000 accessible pMHC products. This estimate does not take into account that the quantitation relies on bivalent primary and secondary antibodies, potentially underestimating the surface levels of pMHC two- to four-fold (which corresponds to 80,000–240,000 complexes). Staining these beads with the TCR tetramer (Fig. 4D) revealed that high densities of pMHC were required for adequate detection, with at least more than 3000 pMHCs on their surface (Fig. 4F). In contrast, staining with Env183/A2 mAb could be achieved at 10-fold lower pMHC density (Fig. 4E,F). This increased sensitivity of the mAb we ascribe to a higher intrinsic binding affinity.

### TCR Tetramers Bind Cells only with High Density of Env_183–191_/HLA-A*02:01 Complexes

**Figure 5:**
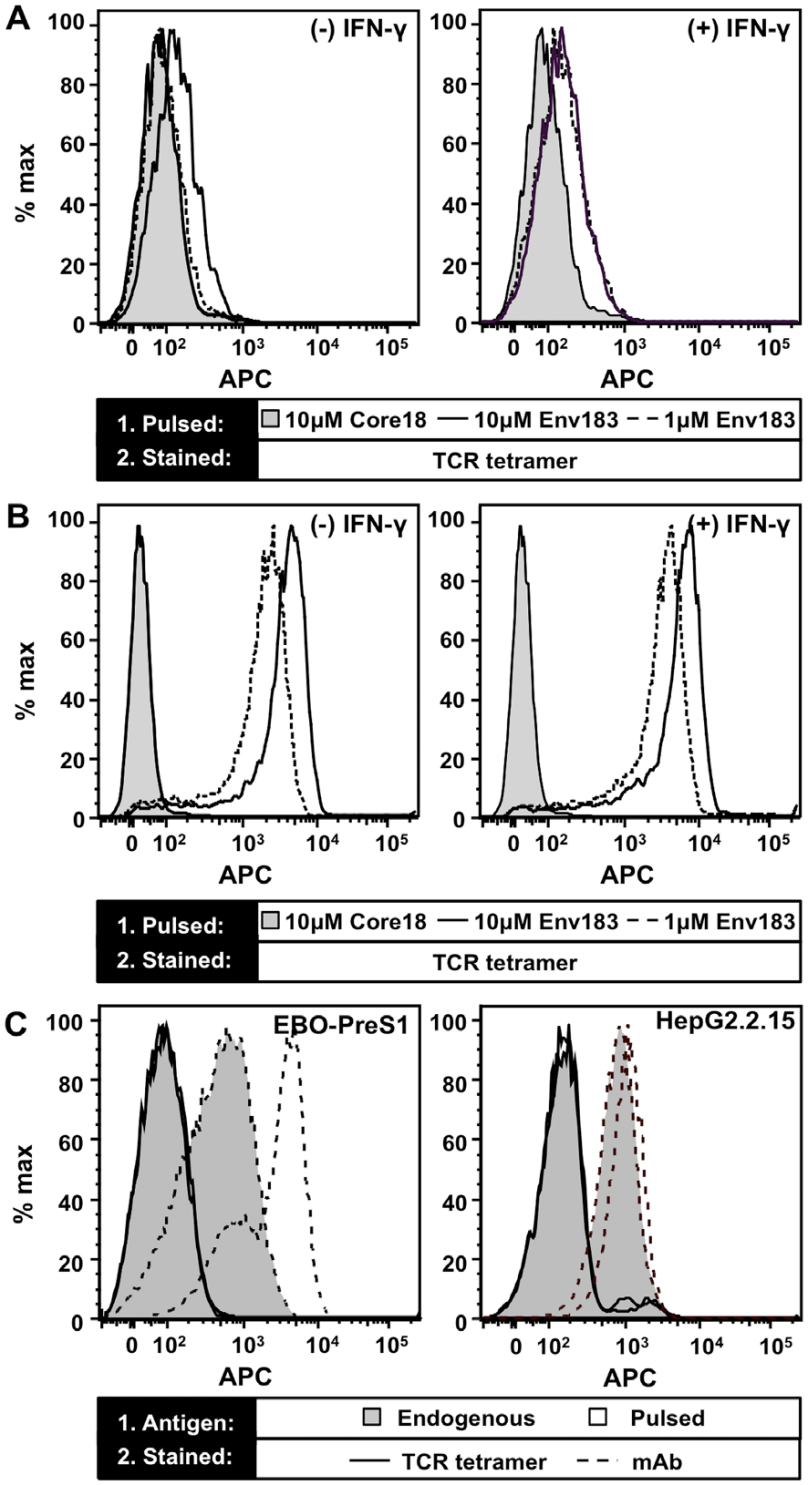
Recognition of pMHC presented on the surface of cells. (A) Untreated (−) and T2 cells treated (+) with IFN-γ were pulsed with 1 or 10 µM of Env_183–191_ or 10 µM of Core_18–27_ peptide control and stained with 1 µg/mL TCR tetramer. (B) Identically treated T2 cells stained with 1 µg/mL Env183/A2 mAb demonstrates that both treatment with IFN-γ and pulsing with higher peptide concentrations results in a higher surface expression of Env183/A2 complexes. (C) HepG2.2.15 and EBO-PreS1 cells, transfected with the full and PreS1 fragment of the HBV genome, respectively, endogenously process and present Env183/A2 pMHC complexes on their cell surface. The mAb (dashed) shows improved detection compared to the TCR tetramers (solid). Peptide pulsed HepG2.2.15 and EBO-PreS1 further increased surface levels of Env183/A2 (filled histograms). Treatment of cells with IFN-γ demonstrably up-regulated HLA-A2 expression (Fig. S5B).

Given that the tetramer of the Env183/A2-specific TCR could bind immobilized pMHC, we next determined if it stained T2 cells (HLA-A*02:01^+^, TAP deficient) pulsed with Env_183–191_ peptide. Monomeric TCRs did not stain peptide-pulsed T2 cells (Fig. S5A), whereas TCR tetramer yielded a moderate but distinct increase in fluorescence specific for the Env183/A2 complex at the highest peptide concentration tested (Fig. 5A). As IFN-γ treatment is known to upregulate the number of MHC molecules on the cell surface (Fig. S5B), the experiment was also performed with T2 cells incubated with IFN-γ followed by pulsing with peptide. IFN-γ treated cells displayed an increase in staining with the TCR tetramer at both 1 and 10 µM Env_183–191_ peptide (Fig. 5A). In contrast, the Env183/A2 mAb yielded a significantly greater shift in fluorescence, which was further increased upon IFN-γ treatment (Fig. 5B). The reduced staining by the TCR tetramer thus suggests that only a fraction of the Env183/A2 complexes on the T2 cells are capable of retaining bound TCR tetramer after washing, reflecting the low affinity of the TCR [20].

Furthermore, we compared the ability of the TCR tetramer and the Env183/A2 mAb to recognize Env183/A2 complexes produced endogenously by HBV-infected cells. We utilized an EBV-immortalized B cell line (EBO-PreS1) transfected with the open-reading frame for the PreS1 fragment of the HBV genome [39], which expresses only the envelope proteins together with the Env_183–191_ epitope. In addition, HepG2.2.15 cells were used, that derive from a stably transfected HLA-A*02:01^+^ hepatoma cell line bearing the complete HBV genome, expressing all viral RNAs and proteins [44]. Both cell lines were treated with IFN-γ before staining. As the EBO-PreS1 and HepG2.2.15 cell lines are both TAP^+^, they present self-peptides in addition to HBV epitopes and the steady state number of Env183/A2 ligands for the Env183/A2 antibody and the TCR tetramer should be considerably lower than peptide-pulsed cells (Fig. 5C). Therefore, at best, minimal staining was observed with TCR tetramers, yet the Env183/A2 mAb stained both cell lines.

The beads with immobilized Env183/A2 complexes at various densities (Fig. 4D) along with the pulsed T2 cells results suggest that although the TCR tetramers bound specifically, the density of the HLA complexes critically influences their binding. Estimates of the density of Env183/A2 on the beads, with a surface area of ∼25 µm^2^, are ∼3200/µm^2^ at their highest concentration. Assuming that an APC expresses 5–10×10^4^ class I MHCs on average [45] which can be pulsed to saturation with Env_183–191_ peptide, a density of 800/µm^2^ can be achieved; which is sufficient only for detection by TCR-like mAb.

## Discussion

T cell activation is preceded by several molecular interactions; the binding of the peptide to an MHC product, and the binding of a TCR to this pMHC complex. In the case of CD8^+^ T cells, activation is also critically dependent on the binding of the CD8 co-receptor to the non-polymorphic regions of the class I MHC product. There has been considerable interest in using soluble extracellular domains of TCRs to probe T cell specificity and pMHC affinity independently from the CD8 binding synergism [46]–[48]. Furthermore, soluble TCRs could in principle serve as reagents to probe surface expression of specific pMHC combinations on cells; particularly in cases where T cell activity assays are challenging (*e.g.* in immunohistological studies). Finally, a novel tumor immunotherapy approach recently demonstrated that such constructs have the ability to target MHC-restricted tumor-associated antigens presented by cancer cells [49]. In this study, the binding and specificity of a hepatitis B virus-specific TCR was examined, and compared to a mAb recognizing the same Env_183–191_/HLA-A*02:01 antigen.

We developed a system to sensitively discriminate the low affinity interactions between the pMHC and the targeting molecules. The assay employs streptavidin-coated beads to immobilize pMHC molecules at high density, similar to what can be achieved with an SPR chip-based approach. When probed with multivalent TCRs or TCR-like mAbs, the bead populations allow rapid determination of peptide fine-specificity by flow cytometry analysis. The bead-assay proved superior to using peptide-pulsed APCs, which was attributed to the high surface density of the antigen (*vide infra*). Furthermore, the polymer beads gave good control over the pMHC surface distribution, which was easily quantified, and allowed analysis of the specific interaction in the absence of adhesion molecules, co-receptors and pMHC products presenting alternative peptides, all of which are abundantly present on APCs.

The bead-based assay revealed that the HBV-specific TCR was properly assembled to recognize its cognate Env183/A2 antigen. Although the enhanced affinity of the TCR-like mAb holds promise for its application as sensitive probe or as targeting therapeutic, it differed from the corresponding TCR in its fine-specificity for the Env183/A2 antigen, most notably at the central 187 residue; a position where viral sequence variation is known to occur. The molecular model that we generated for the Env_183–191_/HLA-A*02:01 complex prominently featured a protruding basic amino acid residue (*i.e.* Arg_187_ for HBV genotypes A/C/D, or Lys_187_ for HBV genotype B) from the binding pocket, and highlighted it as a important candidate residue for interaction with the TCR or the mAb. As the naturally occurring R187K mutation between HBV genotypes A/C/D and genotype B already eliminated binding by the Env183/A2 mAb, the R187A substitution was equally disruptive to binding, as would be predicted [7]. The TCR tetramers, on the other hand, bound both the R187 and K187 epitope variants, although the more profound R187A mutation did interfere with its specific recognition. Structural studies of TCR-like antibodies in complex with their cognate pMHC ligand have shown that both a diagonal binding mode (conventionally employed by TCRs), as well as a non-canonical orientation, can be adopted [50], [51]. Such differences in the chemistries of binding are likely to be at the basis of the distinct fine-specificities of the Env183/A2 mAb and its TCR tetramer counterpart presented here.

The half-life of TCR:pMHC interactions ranges in seconds, whereas oligomerization can extend that by several hundred-fold, thus enabling TCR tetramers to stably bind pMHCs long enough to employ them experimentally as surface staining reagents [15]. The avidity gained through tetramerization of our Env183/A2-specific TCR allowed the tetramers to bind beads as well as the Env183/A2 mAb when saturated with the correct pMHC. The sensitivity of our TCR tetramer staining was clearly dependent on the degree of packing of pMHC on the beads, presumably because at higher density the TCR tetramer can bind clusters of pMHC simultaneously [16], [52], [53]. This reliance of TCR tetramers on the pMHC density has been observed in cellular experiments as well, where the staining intensity decreased proportionally with the titration of cognate pMHC ligand [14]. In our hands, the TCR tetramer showed appreciable staining when loading of the individual beads, with a diameter of ∼2.7 µm, reached above 3,500 pMHC molecules. For a typical hepatocyte, with a diameter of ∼20–40 µm [54], the number of surface pMHCs must be 50-fold higher to attain similar densities. Therefore, to achieve the same level of staining as observed in our bead assay, over 175,000 Env183/A2 complexes would have to be presented. Hepatocytes, however, are poor antigen presenting cells with low expression levels of class I MHC [6] and their capacity for antigen presentation is inefficient [45], making these specific viral pMHC products rare in a clinical setting. With low surface antigen densities, the application of a TCR-like antibody thus appears more practical, even though the TCR tetramer has the capability of binding with similar avidity. A caveat of this estimate for the lower limits of detection, on the other hand, is that it assumes random distribution over membrane, whereas pMHC molecules could conceivably cluster [55]–[57]. It therefore remains to be established if such clustering might allow binding by multivalent TCR constructs.

Collectively, we demonstrated the utility of the bead-based flow cytometry assay in conjunction with the peptide-exchange strategy for pMHC [35]–[38], [41]–[43] to rapidly characterize the antigen-specificity and cross-reactivity of soluble TCRs and TCR-like mAbs, which is of significance especially when the two have demonstrably different docking orientations to their cognate pMHC [51], [58]. Our results also underscore that antigen distribution is an essential parameter to be considered during the development and design of novel diagnostic and immunotherapeutic intervention strategies.

## Supporting Information

Figure S1 **Amino acid sequences and features of the constructs of the Env_183–191_ specific TCR.** Complementarity determining regions (CDRs) of (A) TCRα and (B) TCRβ chains are indicated by black lines; conserved cysteine residues involved in the immunoglobulin-fold disulfide bonds are highlighted in orange; residues mutated into cysteine residues for the non-native interchain disulfide bond are highlighted in blue and the extra cysteine residue in Cβ that was mutated into a serine is shown in green.
(TIF)

Figure S2 **Gel purification profiles.** *In vitro* refolded TCR was purified by size exclusion chromatography (A) followed by anion exchange chromatography (B). Fractions collected, indicated in red boxes, from size exclusion chromatography were pooled and further purified by anion exchange chromatography. Two gradients were used for elution in the anion exchange purification step. A steep 0–15% gradient was followed by a shallow 15–25% gradient of elution buffer (20 mM Tris, pH 8.0 with 1 M NaCl), replacing the binding buffer (20 mM Tris, pH 8.0)
(TIF)

Figure S3 Beads are coated with equivalent levels of Env_183–191_ alanine peptide variants/HLA-A*02:01 complexes. Beads loaded with different Env_183–191_ alanine variant pMHCs were probed stained with a mouse anti-β_2_m antibody followed by detection by an APC-conjugated goat anti-mouse antibody. The mean fluorescent intensities (MFI) indicate that there are equal levels of each pMHC present on the surface of the beads.
(TIF)

Figure S4 **Quantification of pMHC on streptavidin beads.** (A) The QIFI™ quantification kit contains 5 beads conjugated with known number of mouse IgG. IgG. The beads were stained with an APC-conjugated goat anti-mouse and the mean fluorescent intensity (MFI) of each bead population was recorded. A linear regression was determined between the log(MFI) and log(no. of IgG) and the parameters were used for later calculations of pMHC numbers on the streptavidin beads. (B) The bead used in Figure 4 were first stained with a mouse anti-b2m antibody and subsequently with the same APC-conjugated goat anti-mouse antibody used in (A). The mean fluorescence intensity of each bead population and the number of pMHC complexes were calculated based on parameters determined in (A).
(TIF)

Figure S5 **TCR monomers fail to bind peptide pulsed T2 cells and treatment of T2 cells with IFN-γ upregulated expression of MHC.** (A) TCR monomers were used at 1 µg/mL and 5 µg/mL to stain T2 cells pulsed with 10 µM Env_183–191_ peptides. Binding was probed by first incubation with 1 µg/mL of mouse anti-αβTCR antibody followed by an PE-conjugated goat anti-mouse antibody. Monomeric TCRs gave no significant staining. Env183/A2 mAb staining was used as positive control. (B) Untreated and T2 cells treated with 100 U/mL of IFN-γ were stained with an anti-β2m antibody and detected by an APC-conjugated goat anti-mouse antibody, demonstrating that IFN-γ treatment boosts the MHC expression on the surface of T2 cells.
(TIF)
