# Supplementary figures and images for: Binding of TCR Multimers and a TCR-Like Antibody with Distinct Fine-Specificities Is Dependent on the Surface Density of HLA Complexes
Source: PLoS One. 2012 Dec 10;7(12):e51397. doi: 10.1371/journal.pone.0051397 (PMC3519586; doi:10.1371/journal.pone.0051397)

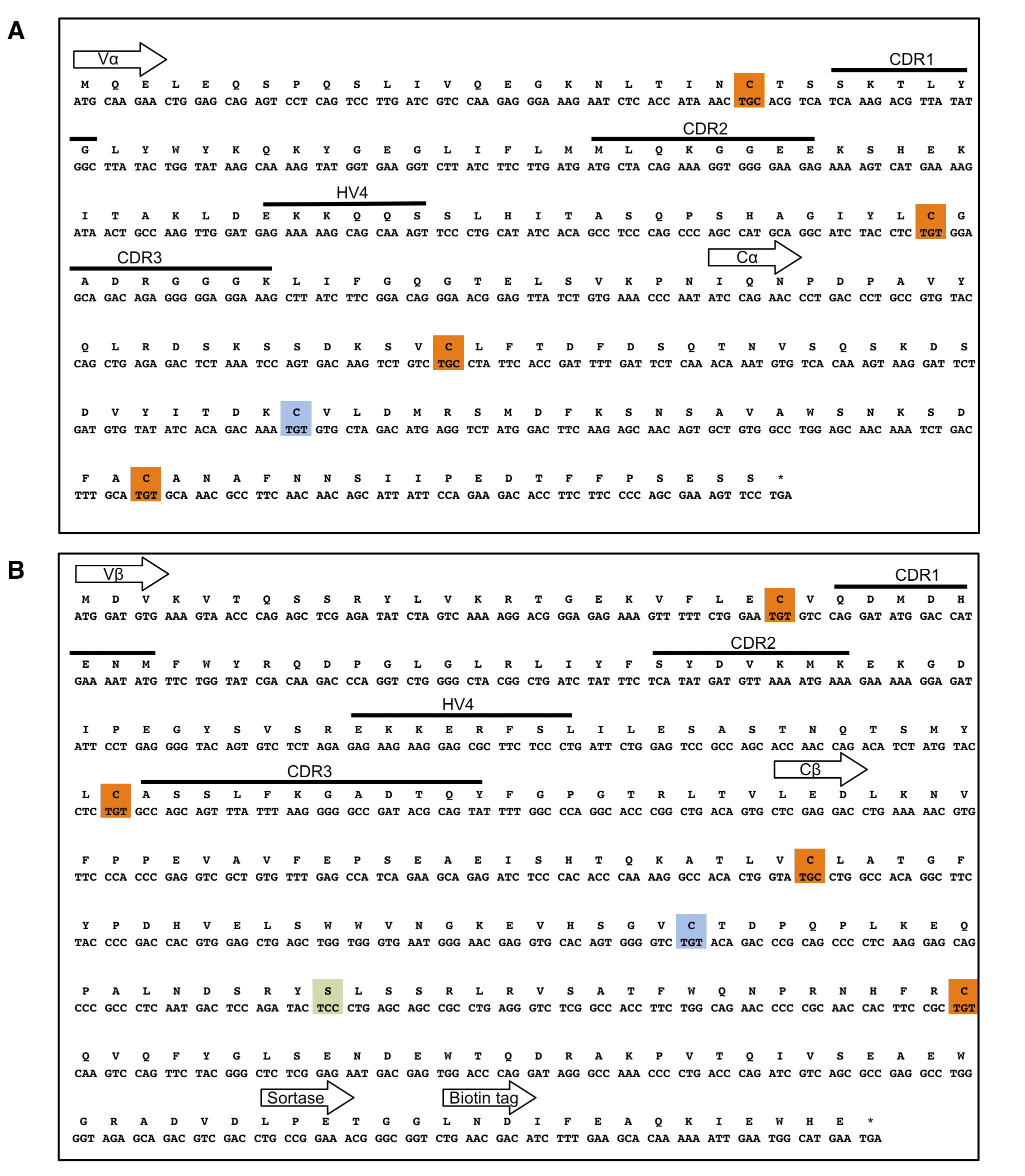

Supplement: Figure S1 — Amino acid sequences and features of the constructs of the Env183–191 specific TCR. Complementarity determining regions (CDRs) of (A) TCRα and (B) TCRβ chains are indicated by black lines; conserved cysteine residues involved in the immunoglobulin-fold disulfide bonds are highlighted in orange; residues mutated into cysteine residues for the non-native interchain disulfide bond are highlighted in blue and the extra cysteine residue in Cβ that was mutated into a serine is shown in green. (TIF) [file pone.0051397.s001.tif]

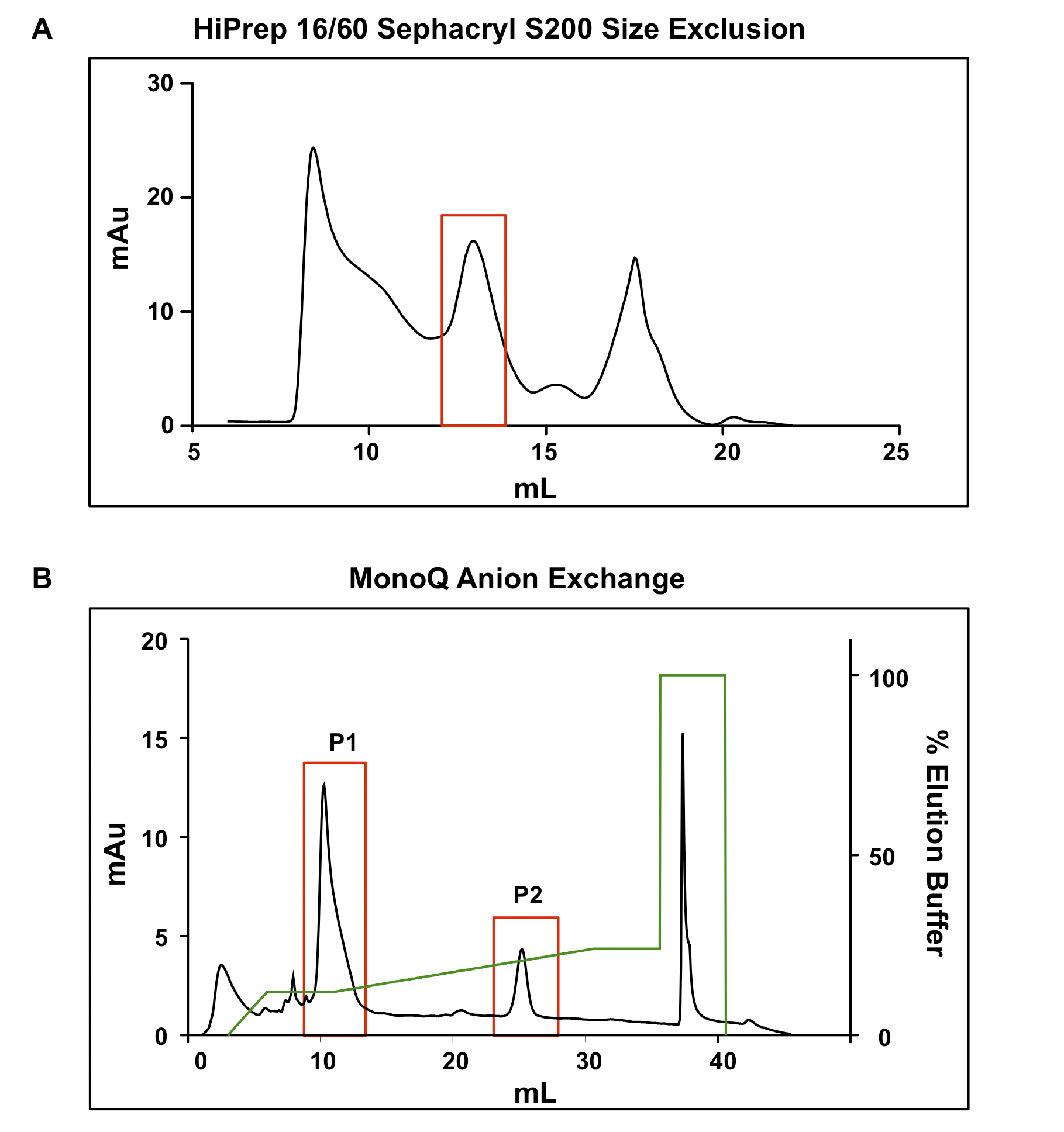

Supplement: Figure S2 — Gel purification profiles. In vitro refolded TCR was purified by size exclusion chromatography (A) followed by anion exchange chromatography (B). Fractions collected, indicated in red boxes, from size exclusion chromatography were pooled and further purified by anion exchange chromatography. Two gradients were used for elution in the anion exchange purification step. A steep 0–15% gradient was followed by a shallow 15–25% gradient of elution buffer (20 mM Tris, pH 8.0 with 1 M NaCl), replacing the binding buffer (20 mM Tris, pH 8.0) (TIF) [file pone.0051397.s002.tif]

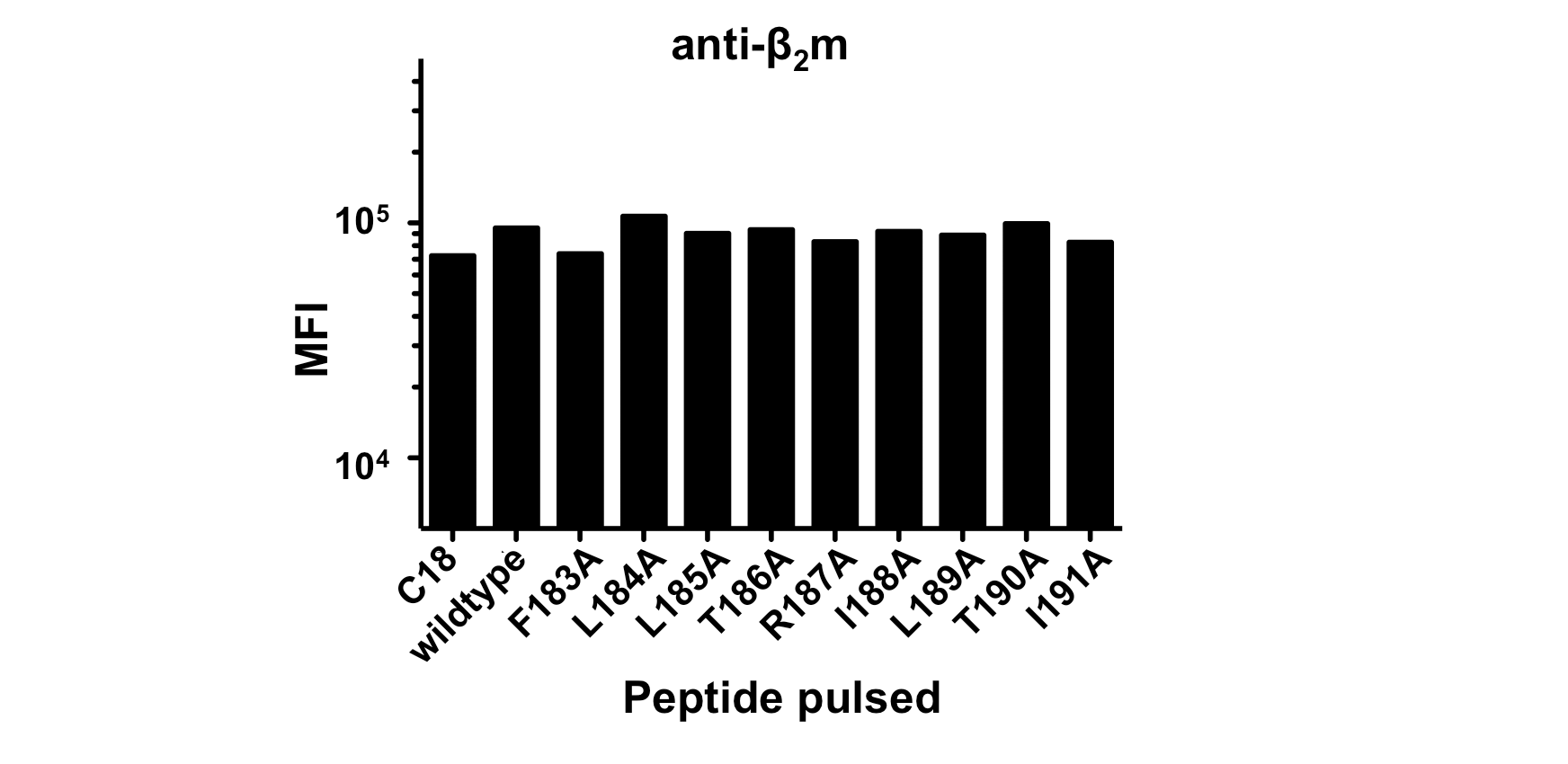

Supplement: Figure S3 — Beads are coated with equivalent levels of Env183–191 alanine peptide variants/HLA-A*02:01 complexes. Beads loaded with different Env183–191 alanine variant pMHCs were probed stained with a mouse anti-β2m antibody followed by detection by an APC-conjugated goat anti-mouse antibody. The mean fluorescent intensities (MFI) indicate that there are equal levels of each pMHC present on the surface of the beads. (TIF) [file pone.0051397.s003.tif]

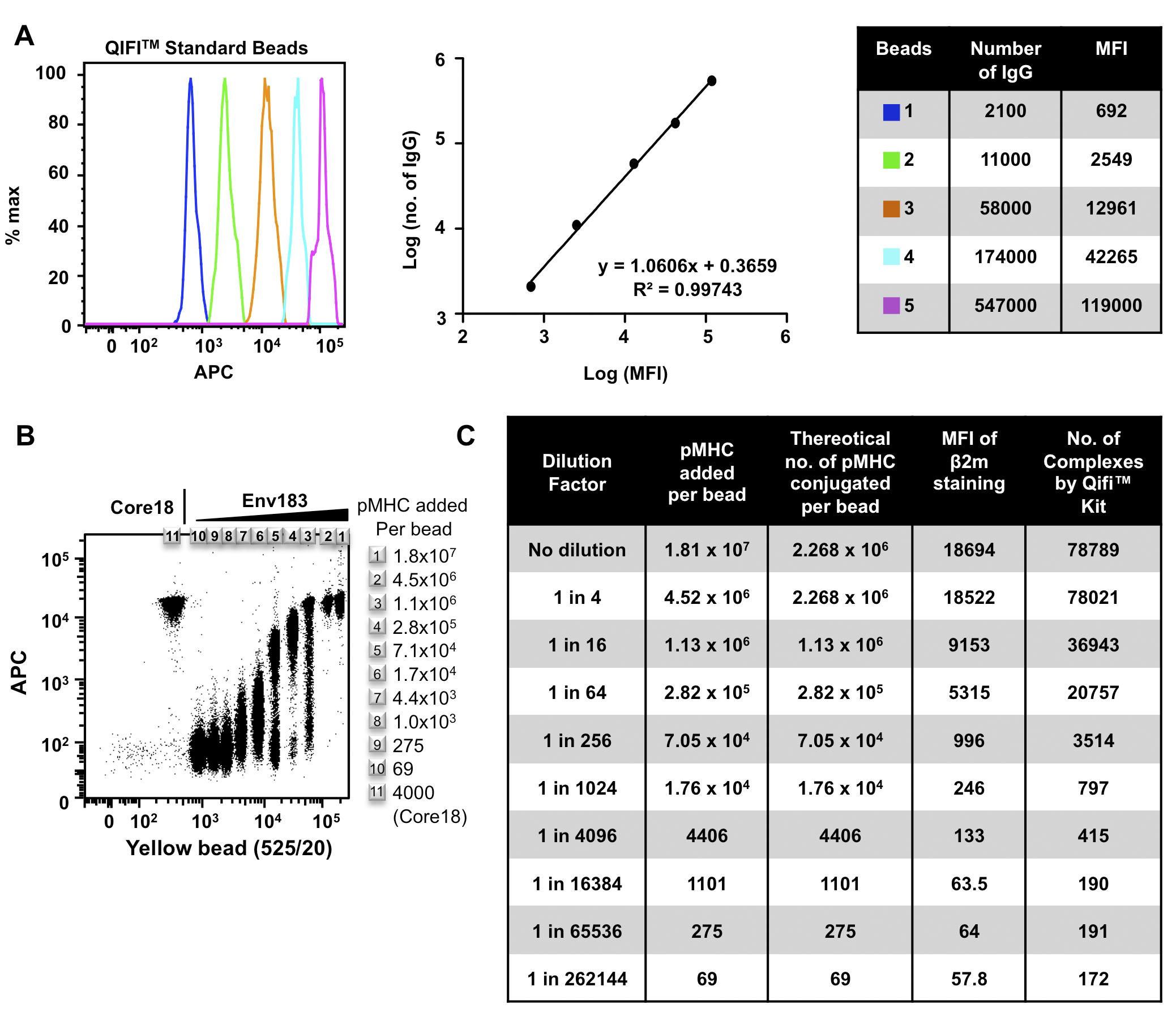

Supplement: Figure S4 — Quantification of pMHC on streptavidin beads. (A) The QIFI™ quantification kit contains 5 beads conjugated with known number of mouse IgG. IgG. The beads were stained with an APC-conjugated goat anti-mouse and the mean fluorescent intensity (MFI) of each bead population was recorded. A linear regression was determined between the log(MFI) and log(no. of IgG) and the parameters were used for later calculations of pMHC numbers on the streptavidin beads. (B) The bead used in Figure 4 were first stained with a mouse anti-b2m antibody and subsequently with the same APC-conjugated goat anti-mouse antibody used in (A). The mean fluorescence intensity of each bead population and the number of pMHC complexes were calculated based on parameters determined in (A). (TIF) [file pone.0051397.s004.tif]

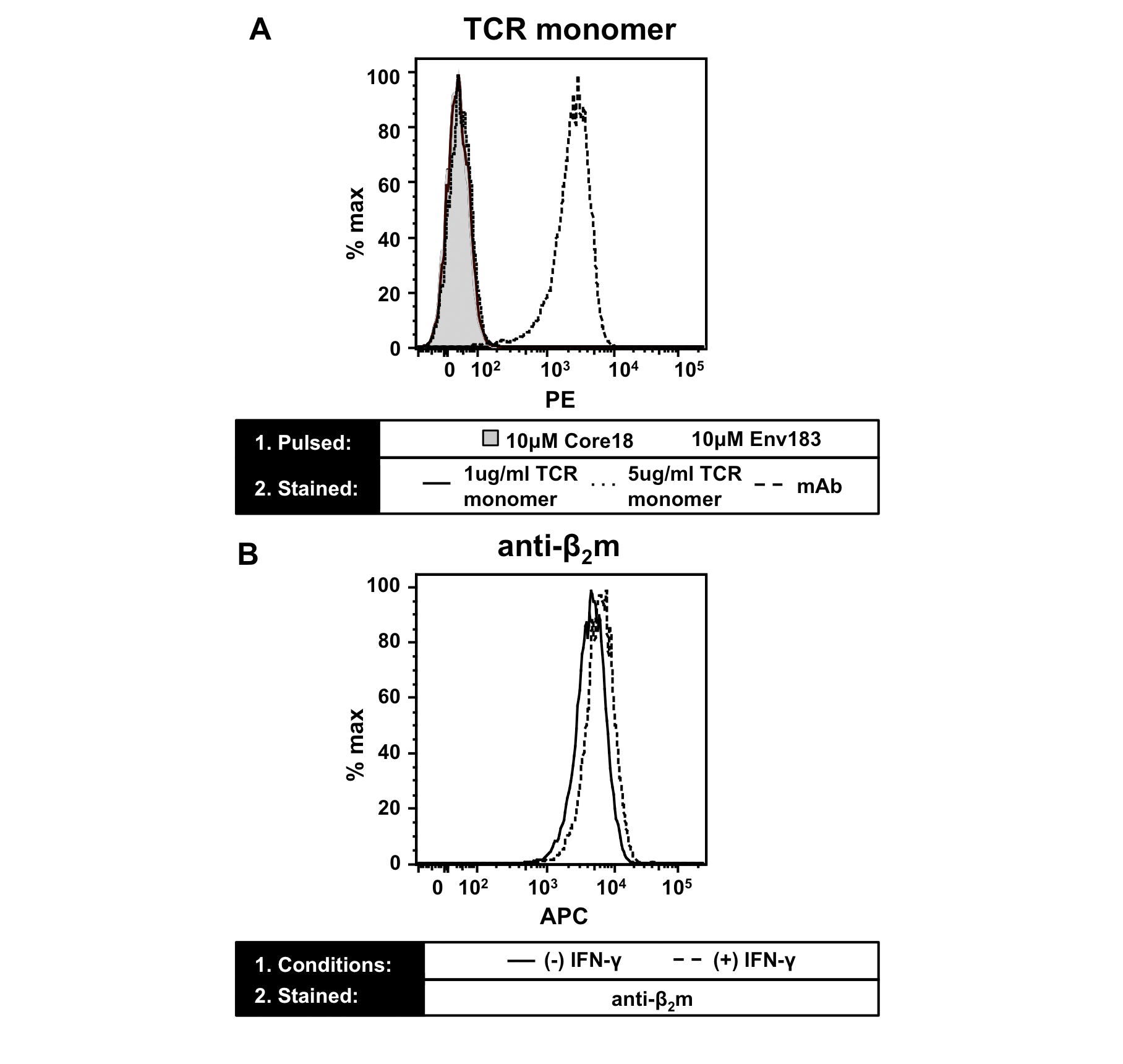

Supplement: Figure S5 — TCR monomers fail to bind peptide pulsed T2 cells and treatment of T2 cells with IFN-γ upregulated expression of MHC. (A) TCR monomers were used at 1 µg/mL and 5 µg/mL to stain T2 cells pulsed with 10 µM Env183–191 peptides. Binding was probed by first incubation with 1 µg/mL of mouse anti-αβTCR antibody followed by an PE-conjugated goat anti-mouse antibody. Monomeric TCRs gave no significant staining. Env183/A2 mAb staining was used as positive control. (B) Untreated and T2 cells treated with 100 U/mL of IFN-γ were stained with an anti-β2m antibody and detected by an APC-conjugated goat anti-mouse antibody, demonstrating that IFN-γ treatment boosts the MHC expression on the surface of T2 cells. (TIF) [file pone.0051397.s005.tif]
